# An Extensive Field Survey Combined with a Phylogenetic Analysis Reveals Rapid and Widespread Invasion of Two Alien Whiteflies in China

**DOI:** 10.1371/journal.pone.0016061

**Published:** 2011-01-21

**Authors:** Jian Hu, Paul De Barro, Hua Zhao, Jia Wang, Francesco Nardi, Shu-Sheng Liu

**Affiliations:** 1 Ministry of Agriculture Key Laboratory of Molecular Biology of Crop Pathogens and Insects, Institute of Insect Sciences, Zhejiang University, Hangzhou, China; 2 CSIRO Ecosystem Sciences, Indooroopilly, Queensland, Australia; 3 Department of Evolution Biology, University of Siena, Siena, Italy; King's College London, United Kingdom

## Abstract

**Background:**

To understand the processes of invasions by alien insects is a pre-requisite for improving management. The whitefly *Bemisia tabaci* is a cryptic species complex that contains some of the most invasive pests worldwide. However, extensive field data to show the geographic distribution of the members of this species complex as well as the invasion by some of its members are scarce.

**Methodology/Principal Findings:**

We used field surveys and published data to assess the current diversity and distribution of *B. tabaci* cryptic species in China and relate the indigenous members to other Asian and Australian members of the complex. The survey covered the 16 provinces where indigenous *B. tabaci* occur and extends this with published data for the whole of China. We used molecular markers to identify cryptic species. The evolutionary relationships between the different Asian *B. tabaci* were reconstructed using Bayesian methods. We show that whereas in the past the exotic invader Middle East-Asia Minor 1 was predominant across China, another newer invader Mediterranean is now the dominant species in the Yangtze River Valley and eastern coastal areas, and Middle East-Asia Minor 1 is now predominant only in the south and south eastern coastal areas. Based on mtCO1 we identified four new cryptic species, and in total we have recorded 13 indigenous and two invasive species from China. Diversity was highest in the southern and southeastern provinces and declined to north and west. Only the two invasive species were found in the northern part of the country where they occur primarily in protected cropping. By 2009, indigenous species were mainly found in remote mountainous areas and were mostly absent from extensive agricultural areas.

**Conclusions/Significance:**

Invasions by some members of the whitefly *B. tabaci* species complex can be rapid and widespread, and indigenous species closely related to the invaders are replaced.

## Introduction

With the rapid increase of world trade and international travel as well as fast economic development in many countries, movement of species by man beyond natural dispersal barriers are happening at an accelerating rate [1]–[4]. Increases of species introductions lead to increased rate of biological invasions, which can have profound negative impact on regional economy and endemic biodiversity [5]–[6]. Impact on endemic biodiversity may happen through intensive competitive interactions between the invaders and their closely related species that occupy similar niches in the ecosystems under invasion, and in many cases rapid displacement of indigenous species by the invaders may occur [7], [8]. While the acceleration of biological invasions in recent years as well as their enormous negative impact has been generally recognized, the processes and patterns of invasions by alien species, especially cryptic species, are poorly understood. In this paper, we show that when extensive field surveys and molecular markers for identifying cryptic species are utilized in an integrated fashion, invasions by alien cryptic species as well as their widespread displacement of indigenous species can be effectively investigated.

The whitefly *Bemisia tabaci* (Gennadius) (Hemiptera: Aleyrodidae) is a cryptic species complex that is globally distributed across much of Africa, southern Europe, the Middle East, the Indian Subcontinent, Asia, Australia, the Pacific and the Americas [9]–[11]. A number of the species that make up the complex are known to damage commercially important plant species either through direct feeding [12] or through the transmission of more than 120 plant viruses primarily belonging to the genus *Begomovirus* (family Geminiviridae) [13], [14].

As there are no morphological characters with which to distinguish the different members of the species complex [15], various molecular methods have been applied to enable the different *B. tabaci* to be separated (see [11] for review). The most widely applied has been mitochondrial cytochrome oxidase 1 (mtCO1). Many of the earlier studies were limited in terms of taxon sampling and identification of the genetic bounds of the various groups being identified [11]. However, the most recent analysis [10] used a more rigorous approach in terms of the breadth of genetic diversity encompassed and the use of pairwise sequence divergence to identify a gap in frequency distribution at 3.5%. This break in the frequency distribution was used to identify the genetic limits of the 24 putative species identified. Furthermore, the analysis developed a set of consensus sequences for these putative species that enabled unidentified mtCO1 sequences to be assigned to a putative species with a high degree of confidence, thereby for the first time creating a rule set with which to identify new species. While reliance on a single gene region to determine species level separation is not without risk, available biological data in the form of reproductive isolation supports the species level separation proposed by Dinsdale et al. [10], [16]–[21]. We have therefore adopted the nomenclature proposed by Dinsdale et al. [10], but have also included the biotype designation so as to enable ready connection to the wider literature.

Most of these putative species have well defined geographic distribution and are regarded as the geographic area's indigenous species [9], [10]. On the other hand, at least two putative species, Middle East - Asia Minor 1 (known commonly as biotypes B and B2; hereon MEAM1) and Mediterranean (known commonly as biotypes Q, J and L; hereon MED) are highly invasive and colonized large areas worldwide. MEAM1 likely originated in the Middle East and Asia Minor regions [18], [22], [23], whereas MED has a distribution that extends from southern Africa to the countries bordering the Mediterranean Sea [9], [10], [18], [24]. MEAM1 most likely invaded China in the mid to late 1990s [25], whereas MED was first detected in China in 2003 [26]. Local field surveys on the distribution and diversity of Chinese members of the species complex have been undertaken for some provinces such as Zhejiang, Shandong, Guangdong, Jiangsu, and Yunnan [7], [27]–[29]. However, no large-scale field survey on *B. tabaci* is available for all of China. We therefore sought to a) determine the current distribution of MEAM1 and MED as well as indigenous members of the *B. tabaci* complex across China; and b) explore the genetic relatedness of indigenous Chinese members of the complex and then to place them within the context of the genetic diversity of this species complex across Asia.

## Materials and Methods

### Whitefly collection and DNA extracti

**Figure 1:**
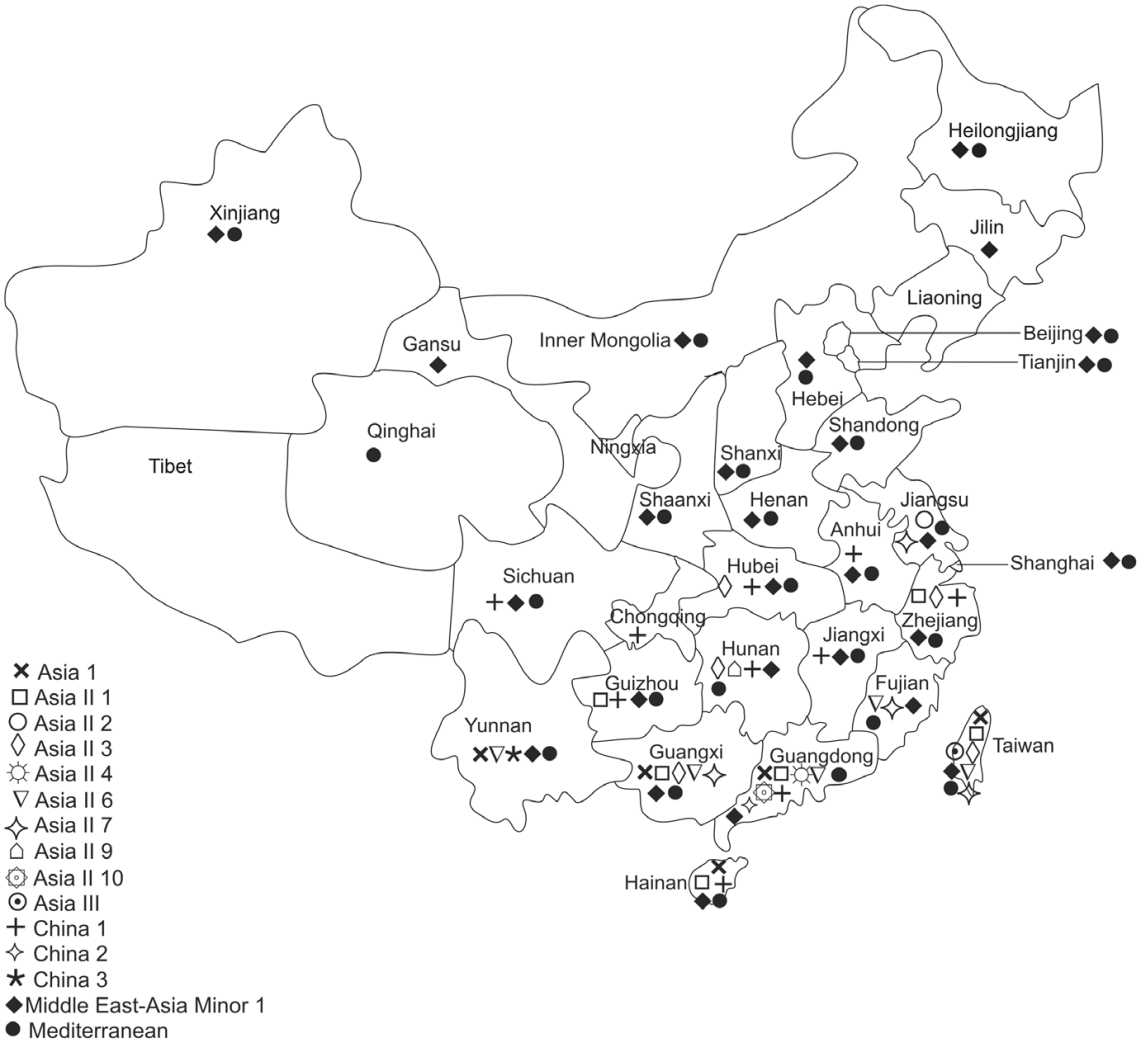
Distributions of the different species belonging to the *B. tabaci* species complex in China. Species records are either from the survey undertaken as part of this study or from records in GenBank (see Fig. 2 for accession details).

**Figure 2:**
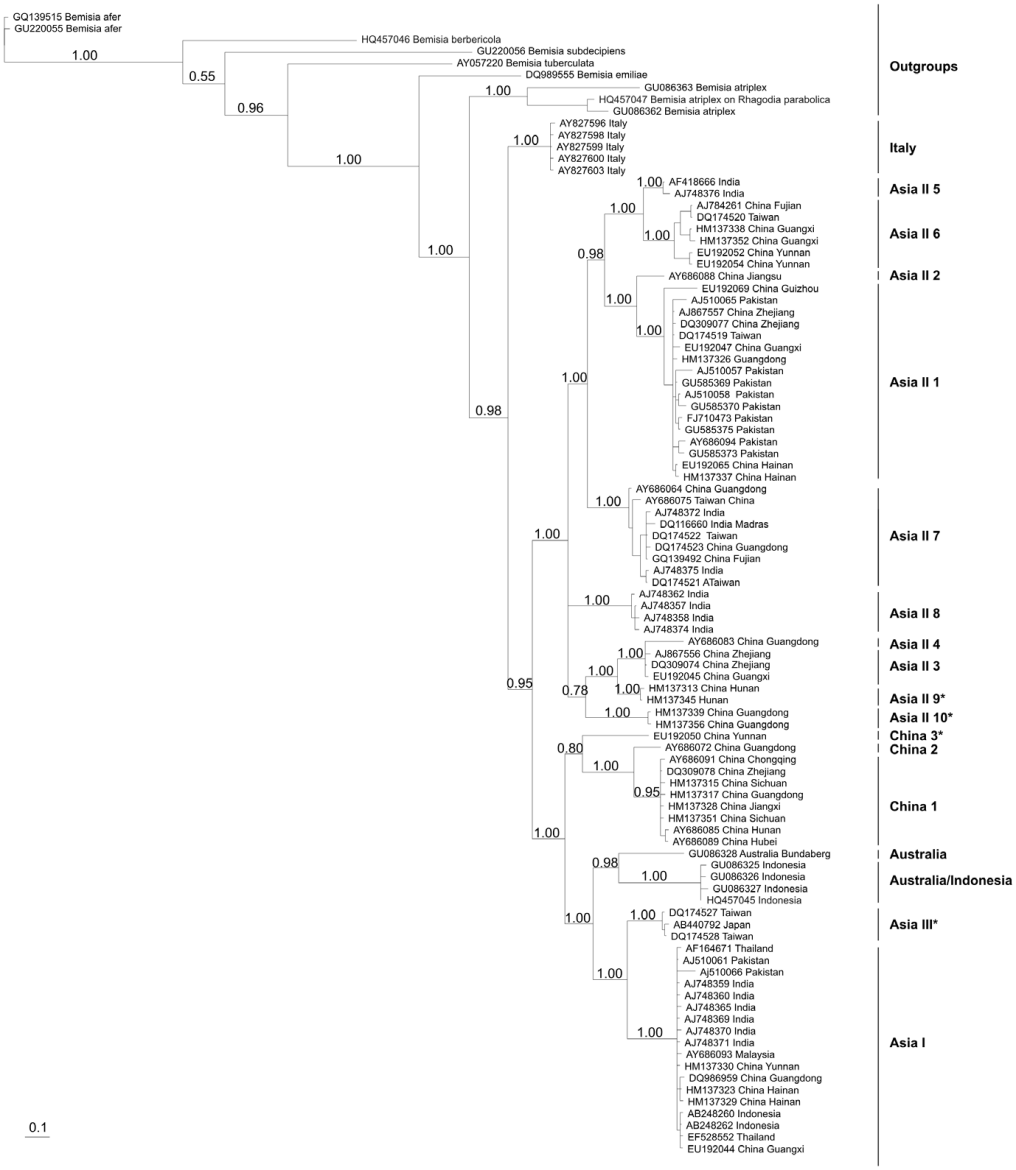
Phylogenetic tree based on the Bayesian analysis of mtCO1 sequences. Posterior probabilities are indicated at nodes. Species indicated by * were identified as being new species in comparison against the consensus sequences from Dinsdale et al. [10] when pairwise sequence divergence exceeded 3.5%.

A large scale sampling of *B. tabaci* was conducted from June 2009 to March 2010 to cover 14 provinces and 2 municipalities (hereon provinces) in east, central, south and southwest of China mainland (Fig. 1). The total area of the 16 provinces is 3.263 million square kilometers and accounts for roughly 1/3 of China mainland. The area is densely populated (approximately 845 million in 2008, or 65% of China total population, People's Republic of China Yearbook 2009) with intensive agriculture, but diverse landscape. Adults were collected from vegetable, ornamental and weed host plant species, and from urban as well as agricultural landscapes. Samples from each of 1–6 different host plants were collected in each of 1–18 sites in each of the provinces. Collection details, geographical locations, host plants and dates of collection are summarized in Table 1 and Table S1. A total of 10,930 whitefly adults were collected. Individual whitefly samples were preserved in 95% ethanol prior to molecular analysis. Total DNA was extracted from individual adult specimens according to De Barro and Driver [30] and Frohlich et al. [22]. DNA was extracted from a maximum of 22 individuals per sample with fewer being used only when <30 whiteflies were available in a sample (as some individuals from each sample had to be retained in our collection). A total of 186 samples containing a total of 3750 individuals were identified to putative species. Voucher specimens are deposited in the collection of the Institute of Insect Sciences at Zhejiang University. In addition, the data was supplemented by records from GenBank (Fig. 2) and the literature to enable records for all mainland provinces as well as the islands of Hainan and Taiwan to be included.

**Table 1:** A summary of the field survey dataset (See Table S1 for details on the collection locations, hosts, collection dates, species and accession numbers of *B. tabaci* mtCO1 sequences).

Provinces	Geographic range (from south to north)	No. of locations/field plots/plant species/plant families surveyed	Collection date	Whitefly species collected
(1) Hainan	18°08′-20°12′N, 108°36′-110°42′E	4/5/3/3	Aug. 2009	MEAM1, Asia I, Asia II 1
(2) Guangdong	20°12′-25°28′N, 109°20′-117°20′E	10/17/11/8	July-Nov. 2009	MEAM1, Asia II 1, Asia II 10, Asia II 6, China 1
(3) Guangxi	21°27′-26°18′N, 104°30′-112°03′E	6/10/7/5	Aug. 2009	MEAM1, MED, Asia II 6
(4) Yunan	21°40′-28°24′N, 97°30′-106°11′E	5/7/7/5	July 2009	MEAM1, MED, Asia I
(5) Fujian	23°30′-28°19′N, 115°51′-120°21′E	7/12/6/4	Nov. 2009	MEAM1,
(6) Jiangxi	24°31′-30°05′N, 113°40′-118°25′E	4/15/12/6	Oct. 2009	MED, China 1
(7) Hunan	24°42′-30°06′N, 108°48′-114°12′E	9/18//10//7	Sept.-Oct. 2009	Med, Asia II 9, China 1
(8) Guizhou	24°45′-29°12′N, 103°40′-109°30′E	2/3/3/3	July 2009	MED, China 1
(9) Zhejiang	27°06′-31°11′N, 118°03′-122°27′E	18/49/15/7	June 2009-Mar. 2010	MEAM1, MED, Asia II 1, Asia II 3, China 1
(10) Sichuan	26°09′-34°12′N, 97°30′-110°06′E	5/5/4/4	July 2009	MEAM1, MED, China 1
(11) Chongqing	28°30′-31°36′N, 105°07′-107°08′E	2/2/2/2	July 2009	China 1
(12) Hubei	29°02′-33°12′N, 108°25′-116°06′E	4/7/6/5	Aug.-Oct. 2009	MED, Aisa II 3, China 1
(13) Anhui	29°23′-34°40′N, 115°26′-119°36′E	5/8/7/5	Oct. 2009	MEAM1, MED
(14) Shanghai	30°41′-31°49′N, 120°58′-121°90′E	1/2/2/2	Nov. 2009	MEAM1, MED
(15) Jiangsu	30°43′-35°07′N, 116°19′-121°54′E	2/7/6/4	Sept. 2009	MED
(16) Henan	31°24′-36°18′N, 110°24′-116°38′E	8/19/12/8	Sept. 2009	MEAM1, MED
Total	18°08′-36°18′N, 97°30′-122°27′E	92/186/29/11	June 2009-Mar. 2010	MEAM1, MED, Asia I, Asia II 1, Asia II 3, Asia II 6, Asia II 9, Asia II 10, China 1

### RAPD-PCR analyses

RAPD-PCR was used as a first screen to identify individuals belonging to the two invasive *B. tabaci* putative species MEAM1 and MED. The methodology followed that of De Barro and Driver [30] using primer H16 (5′-TCTCAGCTGG-3′); this method has proven to be a highly reliable for identifying both MEAM1 and MED and to distinguish them from other members of the complex. Each PCR reaction was performed in a volume of 20 µl containing 2 µl of template DNA, 1.2 U of *Taq* polymerase (Takara, Dalian), 2.5 mM MgCl_2_, 200 µM dNTPs, 2 µM primer, and 2 µl of 10×PCR buffer (Takara, Dalian). PCR procedure consisted of one cycle of 94°C for 5′, 40°C for 2′ and 72°C for 3′, followed by 38 cycles of 94°C for 1′, 40°C for 1′30″ and 72°C for 2′ and a final extension of 72°C for 10′ on a DNA engine PTC-200 Thermocycler (Bio-Rad). Amplification products were separated electrophoretically in 1% agarose gel, and ethidium bromide-stained bands were recorded using a Gel-Doc 2000 system (Bio-Rad). Genotypes corresponding to *B. tabaci* putative species MEAM1 and MED were easily identified based on RAPD banding patterns [17], [30]–[33] and recorded as such. All individuals shown not to belong to MEAM1 or MED were then analysed using mtCO1.

### CO1 gene amplification and sequencing

Three to five individuals from each sample were selected at random for mtCO1 sequencing. Partial CO1 gene sequence (759 bp) was amplified via PCR using universal primers C1-J-2195 (5′-TTGATTTTTTGGTCATCCAGAAGT-3′) and TL2-N-3014 (5′-TCCAATGCACTAATCTGCCATATTA-3′) [34]. Reaction conditions were as above, with a cycle consisting of an initial denaturation of 94°C for 5′, followed by 35 cycles of 94°C for 45″, 50°C for 1′ and 72°C for 1′ 30″, and a final extension of 72°C for 10′. PCR products were gel purified using the Agarose Gel DNA Purification Kit ver. 2.0 (Takara) and directly sequenced on both strands in an ABI 3730 DNA analyzer. A total of 48 new sequences were deposited in GenBank (Accession numbers HM137313 to HM137360).

### Sequence Alignment and Phylogenetic Analysis

The 48 sequences obtained from the above analysis were supplemented with a further 131 sequences from GenBank. The sequences were aligned using ClustalX (ver. 1.81; [35]) and then checked for duplicates, gaps, ambiguous bases, pseudogenes and sequences which were shorter than the required 610 bases starting with 5′GCTATAATAACT. The final dataset contained 88 sequences plus a further 9 outgroup sequences from the species *B. afer*, *B. berbericola*, *B. subdecipiens*, *B. tuberculata*, *B. atriplex*, *B. emiliae* and an undescribed species of *Bemisia* closely related to *B. atriplex* from the host plant *Rhagodia parabolica*. Dinsdale et al. [10] showed that members of the *B. tabaci* species complex could be readily assigned to species by comparison against consensus sequences. They determined that an unknown sequence was a match for a consensus sequence if it diverged by <3.5%; if an unknown sequence diverged by >3.5% from any of the 24 consensus sequences then this was likely to be a new putative species. Some individuals from our samples diverged from all known consensus sequences by >3.5% so a Bayesian phylogenetic analysis following the method used in Dinsdale et al. [10] was undertaken using the MrBayes (ver 3.1; [36]). We partitioned the data using a codon-partition model in which each codon position was allowed its own parameter estimates. The best-fit model of evolution was determined by the Likelihood Ratio Test using Modeltest 3.6 [37]. All partitions were allowed a GTR + invariants + gamma model and analyses were run for 10 million generations by using eight chains and sampling every 1,000 generations. The burn-in period (n = 260) was determined by comparing graphical output from the SUMP command and checking that the harmonic means of the separate runs had converged to within two units. Dinsdale *et al*. [10] showed that the members of the *B. tabaci* complex from Asia formed an extremely well defined monophyletic group. As we were interested only in the relationships between Asian members of the complex, we did not include non-Asian members of the complex. The subsequent consensus tree was visualised using Treeview (Page RDM 2001) and putative species names are those from in Dinsdale et al. [10] except where a new putative species has been identified.

## Results

### Invasive *B. tabaci* species

**Figure 3:**
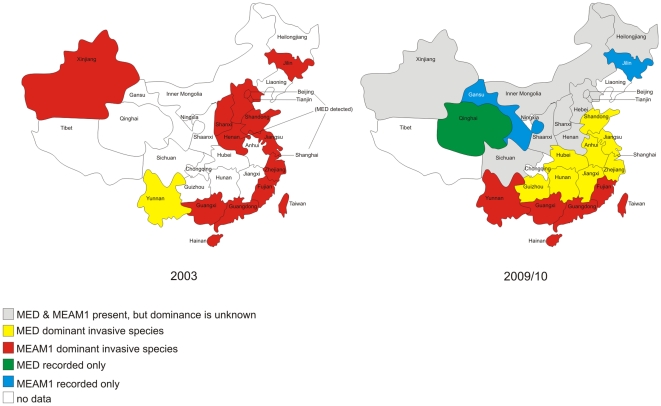
Distribution of the most frequently encountered invasive *B. tabaci* (MEAM1 and MED) in each province in China in 2003 and 2009/10. Data for the distributions in 2003 was obtained from Wu et al. [42], Chu et al. [26], Chu et al. [28] and Hsieh et al. [59]. The distributions for 2009/10 were either derived from this survey or from GenBank accessions.

Our survey and the data from GenBank show that both MEAM1 and MED are widely distributed across China (Table 1, Table S1 and Fig. 1). MEAM1 was found or has been previously recorded from Anhui, Beijing, Fujian, Gansu, Guangdong, Guangxi, Guizhou, Hainan, Hebei, Heilongjiang, Henan, Hubei, Hunan, Inner Mongolia, Jiangsu, Jiangxi, Jilin, Shaanxi, Shandong, Shanghai, Shanxi, Taiwan, Tianjin, Xinjiang, Yunnan and Zhejiang while MED was found in Anhui, Beijing, Fujian, Guangdong, Guangxi, Guizhou, Hainan, Hebei, Heilongjiang, Henan, Hubei, Hunan, Inner Mongolia, Jiangsu, Jiangxi, Qinghai, Shaanxi, Shandong, Shanghai, Shanxi, Taiwan, Tianjin, Xinjiang, Yunnan and Zhejiang (Fig. 1). Only the invasive species occurred in the western and northern provinces north or west of Sichuan and north of Hubei, Anhui and Jiangsu (Fig. 1). Neither MEAM1 nor MED has been recorded from Chongqing, Liaoning, Ningxia and Tibet. When this data is compared to records from samples collected in 2003 or earlier, the shift in dominance from MEAM1 to MED in the provinces of the Yangtze River Valley and eastern coastal areas is apparent. MEAM1 remains dominant in the southern and south eastern coastal areas (Fig. 3).

### Indigenous *B. tabaci* species

Thirteen indigenous whitefly species were found either in the field survey or from records in GenBank (Table 1, Table S1 and Fig. 1). Nine of these represent species previously determined in Dinsdale et al. [10]; the remaining four have pairwise divergences that exceed 3.5% either with the consensus sequences or between themselves and so represent new putative species, which we named as Asia II 9, Asia II 10, China 3 and Asia III, respectively. Asia II 9 was collected from *Ipomoea batatas* in Shaoyang in the province of Hunan and Asia II 10 from *Cucurbita moschata* in Maoming and from *Brassica chinensis* in Zhaoqing in the province of Guangdong. Asia III was from Japan and Taiwan; it was identified from three sequences (AB440792, DQ174527, DQ174528) lodged in GenBank ([38]; Fig. 1). Similarly China 3 was from Yunnan and was identified from another sequence (EU192050) lodged in GenBank.

The distributions of the different indigenous species across China are shown in Fig. 1. Of the indigenous species, China 1 is the most widely distributed being found in 10 provinces across China mainland and the island of Hainan. Six species, Asia II 2, Asia II 4, Asia II 9, Asia II 10, China 2 and China 3 were found in only one province each and Asia III in Taiwan only. No indigenous species were found north of Sichuan, Chongqing, Hubei, Anhui and Jiangsu.

### Phylogenetic analysis

The Bayesian phylogenetic reconstruction based on 97 mtCO1 sequences is shown in Fig. 2. The structure of this Asia group is complex. The basal member of the group is Italy which refers to a distinct species so far known only from Sicily [39]. There are then two large subgroups. The first, the Asia II group of species, collectively spans south-east China, India, Pakistan and as far west as Syria. The second comprises species of Australia, China 1-3, Asia I and Asia III. This latter group has three distinct clusters. Two of these contain species associated either with Australia or with China (China 1-3). The remaining cluster contains Asia I which has a pan-Asia distribution extending from Pakistan and east through China and into south-east Asia whereas Asia III appears to be restricted to the islands of Taiwan and Japan. Genetic distances among the Asian members of *B. tabaci* species complex ranged from 0.051 to 0.187 (Table 2).

**Table 2:** Mean Kimura two-parameter genetic distances between putative species belonging to the Asia group.

		1	2	3	4	5	6	7	8	9	10	11	12	13	14	15	16	17	18
1	Asia I	0																	
2	Asia II_1	0.150	0																
3	Asia II_2	0.130	0.053	0															
4	Asia II_3	0.162	0.135	0.080	0														
5	Asia II_4	0.157	0.116	0.059	0.047	0													
6	Asia II_5	0.169	0.098	0.093	0.136	0.123	0												
7	Asia II_6	0.165	0.114	0.109	0.136	0.131	0.067	0											
8	Asia II_7	0.145	0.095	0.103	0.126	0.122	0.114	0.111	0										
9	Asia II_8	0.143	0.134	0.113	0.121	0.134	0.121	0.137	0.118	0									
10	Asia II_9	0.155	0.124	0.092	0.056	0.076	0.115	0.123	0.108	0.123	0								
11	Asia II_10	0.133	0.128	0.104	0.111	0.112	0.135	0.122	0.114	0.122	0.096	0							
12	Asia III	0.078	0.155	0.132	0.153	0.142	0.165	0.152	0.142	0.153	0.148	0.136	0						
13	China 1	0.145	0.182	0.152	0.182	0.180	0.176	0.174	0.187	0.183	0.164	0.175	0.136	0					
14	China 2	0.115	0.140	0.119	0.147	0.132	0.127	0.141	0.153	0.156	0.138	0.146	0.114	0.138	0				
15	China 3	0.129	0.156	0.136	0.160	0.153	0.144	0.137	0.155	0.149	0.148	0.135	0.121	0.144	0.151	0			
16	Aus/Indo	0.124	0.154	0.132	0.153	0.142	0.138	0.140	0.139	0.144	0.132	0.127	0.125	0.140	0.141	0.051	0		
17	Australia	0.127	0.150	0.124	0.155	0.159	0.150	0.151	0.135	0.130	0.136	0.128	0.121	0.117	0.135	0.111	0.104	0	
18	Italy	0.135	0.136	0.120	0.130	0.135	0.138	0.125	0.131	0.116	0.117	0.131	0.122	0.132	0.129	0.124	0.116	0.132	0

## Discussion

This study for the first time unifies our knowledge of the diversity and distribution of *B. tabaci* across China. China has a considerable diversity; of the 14 Asian putative species identified in Dinsdale et al. [10] only five, Australia, Australia/Indonesia, Asia II 5, Asia II 8 and Italy were not found in China. Applying the rule set devised by Dinsdale et al. [10] we add a further four new species, Asia II 9, Asia II 10, Asia III and China 3. While it may be argued that identification of species using mtCO1 is open to debate, all available biological data in the form of mating studies [17]–[21] support the proposition of Dinsdale et al. [10]. The further analysis in this study increases the number of putative species in the *B. tabaci* species complex from 24 to 28 and suggests that China has a diversity that exceeds that of other major land areas. While the results suggest processes (as yet unknown) that may have acted in China to drive the development of diversity, they may to certain extent reflect the variations in sampling intensity between regions. In the past 10 years there has been considerable interest in China in this species complex, but this effort has not been evenly applied across the whole of China or Asia.

The data also shows that the Asian members of the complex are primarily assorted across one of three dominant lineages. One, the Asia II group of species, extends from Asia Minor through the Indian Subcontinent into China; another, Asia 1, ranges from the Subcontinent through China into south-east Asia; and the third, China 1, is so far known only from China mainland and Hainan. The remaining groups Asia III and Australia form isolated groups with restricted geographic distributions. The ancestral position of Italy is curious. The members of this species all come from Sicily. One interpretation is that this population was introduced to Sicily from Asia via trade activity. However, as far as we know the population in Italy is older than any known so far from Asia which tends to argue against this. An alternative draws upon our knowledge of the evolutionary origin of *B. tabaci*. Here the available data suggest that the most likely origin of *B. tabaci* was sub-Saharan Africa [9], so it is possible that the population in Sicily represents a relic of the wave of spread out of Africa.

The patterns of distribution of indigenous *B. tabaci* across China suggest that most of the diversity is associated with the southern and south eastern parts of China mainland extending from Yunnan around the coast to Guangdong. The diversity then declines to the north, west and east of these provinces. The absence of indigenous *B. tabaci* north and west of Sichuan, Chongqing, Hubei, Anhui and Jiangsu is most likely a consequence of average winter temperatures that fall below zero [40]. The presence of the invasive *B. tabaci* north of this line most likely reflects the capacity of these species to colonize crops grown in greenhouses where they are protected from the below zero temperature conditions of winter [40]. These crops are either not utilized as hosts by the indigenous species or are inaccessible due to their lack of resistance to the insecticides that are regularly applied in this system. The high diversity of indigenous *B. tabaci* in the southern and southeastern coastal parts of China may in part reflect the underlying floral biodiversity which is higher than elsewhere in China in many of these provinces ([41]; Fig. S1).

The survey as well as early data from GenBank shows that both invasive members of the complex were widely distributed in China. Host plant record data indicates that both MEAM1 and MED were found mainly on cultivated Solanaceous and Cucurbitaceous vegetables. While the overall distribution of the two invaders largely overlaps, we observed a considerable disparity in relative abundance in open field crops. Furthermore, the survey data suggests that there has been a considerable change in the relative abundances of MEAM1 and MED which suggests that MED is displacing the earlier invader MEAM1. Liu et al. [7] and the review of De Barro et al. [11] have described not only the processes by which invasive *B. tabaci* displace indigenous *B. tabaci*, but also indicate that displacement can take place rapidly, e.g. in Australia MEAM1 displaced the indigenous Australian species in less than five years. As displacement occurs so rapidly we believe that historical data may not reflect current distributions and so we will concentrate our discussion of *B. tabaci* in China on the results from the survey.

MED was the predominant species in the Yangtze River Valley and eastern coastal areas (Fig. 1) while MEAM1 was predominant in the south and south eastern coastal areas (Fig. 1). Wu et al. [42], Zhang et al. [43], Chu et al. [26] and Qiu et al. [44] showed that in 2003 MEAM1 was the only invasive whitefly found in Guangdong, Fujian, Zhejiang, Jiangsu, Jilin (first record 2001), Shandong, Shanghai, Hebei, Beijing, Shanxi, Henan and Xinjing whereas MED was found in Yunnan and Beijing only. MEAM1 is believed to have invaded China in the mid-late 1990s [25] whereas MED was first detected in 2003 from ornamental plants in the Yunnan province [26]; the invasion and subsequent spread of both is via the movement of infested plant material, particularly ornamentals [45]–[47]. Since then, MED has spread over much of China and steadily displaced MEAM1. For example, in Hubei and Shandong between 2005 and 2008 MED has increased from its first detection in 2005 to being predominant by 2008 [47], [48]. Similarly in Zhejiang, MED has displaced MEAM1 in many locations [49]. The capacity for MED to displace MEAM1 would appear to being facilitated by the higher level of insecticide resistance in MED [50]–[52]. For example Luo et al. [53] observed that while MEAM1 from China remained largely susceptible to acetamiprid, imidacloprid, and thiamethoxam, MED from China expressed 20-170 fold resistance. The effects of differential resistance to insecticides on these two invaders has been shown by Crowder et al. [54], [55] to be a key element in MED′s capacity to displace MEAM1, which in situations where insecticide pressure is minimal would be expected to displace MED [55], [56]. Together, these data suggest that as long as current patterns of insecticide use in China are maintained, MED will continue to spread and displace MEAM1. This will presumably have implications for pest management.

Delatte et al. [57] observed that both MEAM1 and MED were most widely distributed in areas characterized by intense horticulture. Our findings are similar. The invaders dominated in intense farming landscapes where Solanaceae and Cucurbitaceae crop hosts are most abundant. In contrast, indigenous species occurred more frequently in areas of less intense, small plot farming activity where they were observed to feed on hosts such as *Ipomoea batatas*, *Glycine max*, and *Humulus japonicas* (Table S1).

Our results and those of Ma et al. [58] indicate that in the provinces of Guizhou and Sichuan, invasive *B. tabaci* were repeatedly detected on ornamental plants in protected culture and very occasionally on cultivated field crop hosts and weeds. This situation has been observed earlier e.g. in Australia and the USA where the earliest detections were also associated with ornamentals under protected culture and only later on field crops (De Barro PJ unpublished data). This suggests that the spread of these species to field crops is only a matter of time.

The data and analyses presented here, apart from providing an extensive picture of *B. tabaci* diversity in China at present, could be used in the future as background information to help monitor the spread of the exotic invaders and their displacement of local species. The patterns of spread and impacts on species diversity will provide useful insights into the invasion process. Furthermore, a clear understanding on the underlying shifts in invader predominance may give greater clarity to the consequences of crop management decisions.

## Supporting Information

Figure S1 **Floral diversity in each province of China based on data from Flora of China (2007), and diversity is presented as the number of plant species per 10 000 km^2^.**
(DOC)

Table S1 **Survey collection locations, hosts, collection dates, species and accession numbers of** ***Bemisia tabaci*** **mtCO1 sequences.**
(DOC)
