# Supplementary material for: An Extensive Field Survey Combined with a Phylogenetic Analysis Reveals Rapid and Widespread Invasion of Two Alien Whiteflies in China
Source: PLoS One. 2011 Jan 21;6(1):e16061. doi: 10.1371/journal.pone.0016061 (PMC3025023; doi:10.1371/journal.pone.0016061)
Supplement: Figure S1 — Floral diversity in each province of China based on data from Flora of China (2007), and diversity is presented as the number of plant species per 10 000 km2. (DOC) [file pone.0016061.s001.doc]

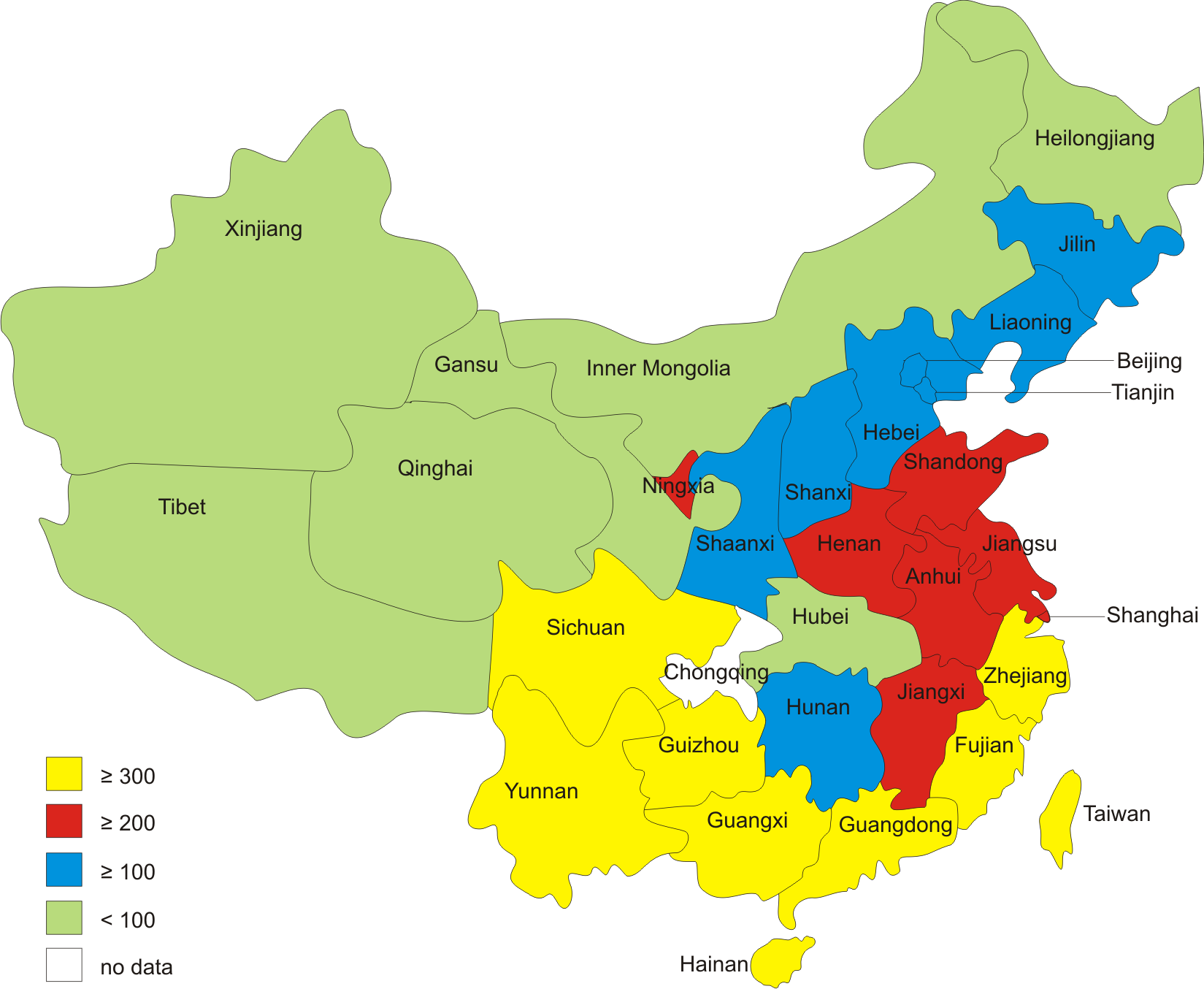


**Fig. S1.** Floral diversity in each province of China based on data from Flora of China (2007), and diversity is presented as the number of plant species per 10 000 km^2^.
