# Supplementary material for: An Extensive Field Survey Combined with a Phylogenetic Analysis Reveals Rapid and Widespread Invasion of Two Alien Whiteflies in China
Source: PLoS One. 2011 Jan 21;6(1):e16061. doi: 10.1371/journal.pone.0016061 (PMC3025023; doi:10.1371/journal.pone.0016061)
Supplement: Table S1 — Survey collection locations, hosts, collection dates, species and accession numbers of Bemisia tabaci mtCO1 sequences. (DOC) [file pone.0016061.s002.doc]

**Table S1** Survey collection locations, hosts, collection dates, species and accession numbers of *Bemisia tabaci* mtCO1 sequences.

| Location* | Latitude | Longitude | Host plant (family) † | Collection date | Whitefly species | GenBank accession no. |
| --- | --- | --- | --- | --- | --- | --- |
| **(1) Hainan province** | | |  |  |  |  |
| Dongfeng, Sanya | 18°24'N | 109°42'E | *Ipomoea batatas* (5) | Aug. 2009 | Asia II 1 | HM137354 |
| Taiping, Wuzhishan | 18°48'N | 109°31'E | *Ipomoea batatas* (5) | Aug. 2009 | Asia II 1 | HM137337 |
| Heiqunpo, Haikou | 19°81'N | 110°52'E | *Ipomoea batatas* (5) | Aug. 2009 | Asia II 1 | HM137335 |
|  |  |  | *Solanum melongena* (2) | Aug. 2009 | MEAM1 |  |
| Longtou, Haikou | 20°01'N | 110°17'E | *Manihot esculenta* (7) | Aug. 2009 | Asia I | HM137329, HM137323 |
| **(2) Guangdong province** | | |  |  |  |  |
| Huguang, Zhanjiang | 21°11'N | 110°14'E | *Glycine max* (4) | Aug. 2009 | China 1 | HM137317 |
| Hongjiang, Zhanjiang | 21°37'N | 109°51'E | *Glycine max* (4) | Aug. 2009 | Asia II 1 | HM137322 |
|  |  |  | *Manihot esculenta* (7) | Aug. 2009 | Asia II 1 | HM137319, HM137326 |
| Henan, Maoming | 21°63'N | 110°86'E | *Ipomoea batatas* (5) | Aug. 2009 | Asia II 6 | HM137342 |
| Kansha, Maoming | 21°93'N | 110°69'E | *Cucurbita moschata* (1) | Aug. 2009 | Asia II 10  China 1  MEAM1 | HM137356 HM137357 HM137358 |
| Longgang, Shenzhen | 22°71'N | 114°24'E | *Cucumis sativus* (1) | Jul. 2009 | MEAM1 |  |
|  |  |  | *Solanum melongena* (2) | Jul. 2009 | MEAM1 |  |
| Modiesha, Guangzhou | 23°06'N | 113°20'E | *Solanum melongena* (2) | Aug. 2009 | MEAM1 |  |
|  |  |  | *Ipomoea batatas* (5) | Aug. 2009 | MEAM1 | HM137314 |
|  |  |  | *Luffa aegyptiaca* (1) | Aug. 2009 | MEAM1 |  |
| Guangzhou botanical garden | 23°19'N | 113°36'E | *Peperomia obtusifolia* (10) | Jul. 2009 | MEAM1 |  |
|  |  |  | *Duranta repens* (11) | Jul. 2009 | MEAM1 |  |
| Zhongluotan, Guangzhou | 23°38'N | 113°44'E | *Cucurbita moschata* (1) | Nov. 2009 | MEAM1 |  |
| Shijian, Zhaoqing | 23°50'N | 112°45'E | *Solanum melongena* (2) | Jul. 2009 | MEAM1 |  |
|  |  |  | *Solanum lycopersicum* (2) | Jul. 2009 | MEAM1 |  |
| Liang village, Zhaoqing | 23°95'N | 112°01'E | *Solanum melongena* (2) | Jul. 2009 | MEAM1 |  |
|  |  |  | *Brassica campestris* ssp. *chinensis* (3) | Jul. 2009 | Asia II 1  Asia II 10 | HM137339 HM137340 |
| **(3) Guangxi Zhuang Autonomous Region** | | |  |  |  |  |
| Gaode, Beihai | 21°27'N | 109°09'E | *Ipomoea batatas* (5) | Aug. 2009 | Asia II 6 | HM137352, HM137353 |
| Malan, Beihai | 21°29'N | 109°09'E | *Ipomoea batatas* (5) | Aug. 2009 | MED | HM137360 |
| Nama, Naning | 22°38'N | 108°23'E | *Vigna unguiculata* (4) | Aug. 2009 | MEAM1 |  |
|  |  |  | *Solanum melongena* (2) | Aug. 2009 | MEAM1 |  |
|  |  |  | *Gossypium hirsutum* (6) | Aug. 2009 | MEAM1 |  |
|  |  |  | *Cucumis sativus* (1) | Aug. 2009 | MEAM1 |  |
| Wutang, Nanning | 22°94'N | 108°54'E | *Luffa aegyptiaca* (1) | Aug. 2009 | MEAM1 |  |
| Naji, Baise | 23°45'N | 106°47'E | *Benincasa hispida* (1) | Aug. 2009 | MEAM1 |  |
| Dawan, Baise | 23°52'N | 106°37'E | *Luffa aegyptiaca* (1) | Aug. 2009 | Asia II 6  MEAM1 | HM137338 |
|  |  |  | *Vigna unguiculata* (4) | Aug. 2009 | MEAM1 |  |
| **(4) Yunnan province** | | |  |  |  |  |
| Yuxi suburbs | 24°35'N | 102°54'E | *Glycine max* (4) | Jul. 2009 | Asia I | HM137332 |
| Ganchong, Honghe | 24°38'N | 103°46'E | *Vigna unguiculata* (4) | Jul. 2009 | Asia I | HM137330, HM137336 |
|  |  |  | *Ipomoea batatas* (5) | Jul. 2009 | Asia I | HM137327 |
| Xuefu, Kunming | 25°06'N | 102°70'E | *Euphorbia pulcherrima* (7) | Jul. 2009 | MED | HM137334 |
| Chuxiong suburbs | 25°69'N | 101°87'E | *Cucumis sativus* (1) | Jul. 2009 | MEAM1 |  |
| Xiaobinglin, Chuxiong | 25°81'N | 101°87'E | *Cucurbita moschata* (1) | Jul. 2009 | MEAM1 |  |
|  |  |  | *Capsicum annuum* (2) | Jul. 2009 | MEAM1 |  |
| **(5) Fujian province** | | |  |  |  |  |
| Qinjiao, Xiamen | 24°47'N | 117°96'E | *Ipomoea batatas* (5) | Nov. 2009 | MEAM1 | HM137325 |
| Qunyao, Xiamen | 24°47'N | 117°97'E | *Brassica oleracea* var. *capitata* (3) | Nov. 2009 | MEAM1 |  |
|  |  |  | *Cucurbita moschata* (1) | Nov. 2009 | MEAM1 |  |
| Jiangshan, Longyan | 25°17'N | 116°98'E | *Cucumis sativus* (1) | Nov. 2009 | MEAM1 |  |
|  |  |  | *Solanum lycopersicum* (2) | Nov. 2009 | MEAM1 |  |
|  |  |  | *Brassica oleracea* var. *capitata* (3) | Nov. 2009 | MEAM1 |  |
|  |  |  | *Solanum melongena* (2) | Nov. 2009 | MEAM1 |  |
| Duwu, Fuzhou | 26°12'N | 119°24'E | *Solanum melongena* (2) | Nov. 2009 | MEAM1 |  |
| Xiqin, Nanping | 26°35'N | 118°43'E | *Brassica oleracea* var. *capitata* (3) | Nov. 2009 | MEAM1 |  |
| Xiadao, Nanping | 26°57'N | 118°27'E | *Cucumis sativus* (1) | Nov. 2009 | MEAM1 |  |
|  |  |  | *Cucumis sativus* (1) | Nov. 2009 | MEAM1 |  |
| Hougu, Nanping | 26°60'N | 118°16'E | *Solanum melongena* (2) | Nov. 2009 | MEAM1 |  |
| **(6) Jiangxi province** | | |  |  |  |  |
| Nashan, Jinggangshan | 26°74'N | 114°30'E | *Ipomoea batatas* (5) | Oct. 2009 | China 1 | HM137328 |
| Sanjiang, Nanchang | 28°29'N | 116°01'E | *Cucumis sativus* (1) | Oct. 2009 | MED |  |
|  |  |  | *Capsicum annuum* (2) | Oct. 2009 | MED |  |
|  |  |  | *Citrullus lanatus* (1) | Oct. 2009 | MED |  |
|  |  |  | *Raphanus sativus* var. *longipinnatus* (3) | Oct. 2009 | MED |  |
| Yangzizhou, Nanchang | 28°72'N | 115°91'E | *Cucurbita moschata* (1) | Oct. 2009 | MED |  |
|  |  |  | *Ipomoea batatas* (5) | Oct. 2009 | MED |  |
|  |  |  | *Brassica campestris* ssp. *pekinensis* (3) | Oct. 2009 | MED |  |
|  |  |  | *Humulus japonicus* (9) | Oct. 2009 | MED |  |
| Yong'an, Jiujiang | 29°76'N | 115°79'E | *Capsicum annuum*(2) | Oct. 2009 | MED |  |
|  |  |  | *Ipomoea batatas* (5) | Oct. 2009 | MED |  |
|  |  |  | *Cucumis sativus* (1) | Oct. 2009 | MED |  |
|  |  |  | *Solanum melongena* (2) | Oct. 2009 | MED |  |
|  |  |  | *Phaseolus vulgaris* (4) | Oct. 2009 | MED |  |
|  |  |  | *Brassica oleracea* var. *capitata* (3) | Oct. 2009 | MED |  |
| **(7) Hunan province** | | |  |  |  |  |
| Chetou, Yongzhou | 25°37'N | 111°34'E | *Ipomoea batatas* (5) | Sep. 2009 | China 1 | HM137343 |
| Bamutian, Yongzhou | 25°57'N | 111°39'E | *Ipomoea batatas* (5) | Sep. 2009 | China 1 | HM137348 |
| Longhui, Shaoyang | 27°17'N | 110°99'E | *Ipomoea batatas* (5) | Oct. 2009 | China 1  Asia II 9 | HM137347  HM137313, HM137345 |
| Longhua, Changsha | 28°11'N | 113°06'E | *Solanum melongena* (2) | Sep. 2009 | MED |  |
|  |  |  | *Ipomoea batatas* (5) | Sep. 2009 | MED |  |
|  |  |  | *Capsicum annuum* (2) | Sep. 2009 | MED |  |
|  |  |  | *Brassica campestris* ssp. *pekinensis* (3) | Sep. 2009 | MED |  |
| Kaifu, Changsha | 28°14'N | 112°59'E | *Cucurbita moschata* (1) | Sep. 2009 | MED |  |
|  |  |  | *Solanum melongena* (2) | Sep. 2009 | MED |  |
|  |  |  | *Capsicum annuum* (2) | Sep. 2009 | MED |  |
| Qingshan, Changsha | 28°23'N | 112°90'E | *Euphorbia pulcherrima* (7) | Sep. 2009 | MED |  |
| Aiban, Jishou | 28°18'N | 109°38'E | *Cucumis sativus* (1) | Sep. 2009 | MED |  |
|  |  |  | *Vigna unguiculata* (4) | Sep. 2009 | MED |  |
|  |  |  | *Raphanus sativus* var. *longipinnatus* (3) | Sep. 2009 | MED |  |
| Xinzhou, Yueyang | 29°26'N | 112°57'E | [*Gossypium hirsutum*](http://en.wikipedia.org/wiki/Gossypium_hirsutum) (6) | Sep. 2009 | MED |  |
| Junshan, Yueyang | 29°30'N | 112°53'E | *Cucurbita moschata* (1) | Sep. 2009 | MED |  |
|  |  |  | *Solanum melongena* (2) | Sep. 2009 | MED |  |
|  |  |  | [*Gossypium hirsutum*](http://en.wikipedia.org/wiki/Gossypium_hirsutum) (6) | Sep. 2009 | MED |  |
| **(8) Guizhou province** | | |  |  |  |  |
| Qingxi, Guiyang | 26°40'N | 106°67'E | *Glechoma longituba* (8) | Jul. 2009 | MED |  |
| Zhennong, Zunyi | 27°39'N | 107°70'E | *Ipomoea batatas* (5) | Jul. 2009 | China 1 | HM137355 |
|  |  |  | *Solanum melongena* (2) | Jul. 2009 | China 1 |  |
| **(9) Zhejiang province** | | |  |  |  |  |
| Cangnan, Wenzhou | 27°49'N | 120°39'E | *Solanum lycopersicum* (2) | Mar. 2010 | MEAM1 |  |
| Ruian, Wenzhou | 27°76'N | 120°64'E | *Solanum lycopersicum* (2) | Feb. 2010 | MEAM1 |  |
| Wencheng, Wenzhou | 27°79'N | 120°09'E | *Ipomoea batatas* (5) | Oct. 2009 | Asia II 1 |  |
|  |  |  | *Solanum melongena* (2) | Oct. 2009 | MEAM1 |  |
|  |  |  | *Brassica oleracea* var. *capitata (3)* | Oct. 2009 | MEAM1 |  |
| Wenzhou Suburbs | 27°97'N | 120°63'E | *Solanum lycopersicum* (2) | Oct. 2009 | MEAM1 |  |
|  |  |  | *Cucumis sativus* (1) | Oct. 2009 | MEAM1 |  |
|  |  |  | *Brassica oleracea* var. *capitata* (3) | Oct. 2009 | MEAM1 |  |
|  |  |  | *Solanum lycopersicum* (2) | Mar. 2010 | MEAM1 |  |
| Jingning, Lishui | 27°97'N | 119°63'E | *Capsicum annuum* (2) | Jun. 2009 | MED |  |
|  |  |  | *Solanum nigrum* (2) | Jun. 2009 | MED |  |
| Longquan, Lishui | 28°08'N | 119°110'E | *Glycine max* (4) | Jun. 2009 | China 1 | HM137349 |
| Suichang, Lishui | 28°56'N | 119°23'E | *Glycine max* (4) | Nov. 2009 | Asia II 1 |  |
| Keqiao, Shaoxing | 28°84'N | 121°11'E | *Solanum lycopersicum* (2) | Nov. 2009 | MEAM1, MED |  |
|  |  |  | *Capsicum annuum* (2) | Nov. 2009 | MED |  |
|  |  |  | *Cucumis sativus* (1) | Nov. 2009 | MED |  |
| Jinhua Suburbs | 29°05'N | 119°68'E | *Gossypium hirsutum* (6) | Nov. 2009 | MED |  |
|  |  |  | *Glycine max* (4) | Nov. 2009 | MED |  |
|  |  |  | *Brassica campestris* ssp. *pekinensis* (3) | Nov. 2009 | MED |  |
| Ninghai, Ningbo | 29°24'N | 121°47'E | *Cucumis sativus* (1) | Nov. 2009 | MED |  |
|  |  |  | *Solanum lycopersicum* (2) | Nov. 2009 | MED |  |
|  |  |  | *Capsicum annuum* (2) | Nov. 2009 | MED |  |
|  |  |  | *Cucurbita pepo* (1) | Nov. 2009 | MED |  |
| Jiande, Hangzhou | 29°45'N | 119°20'E | *Ipomoea batatas* (5) | Nov. 2009 | Asia II 1, China 1 |  |
|  |  |  | *Cucurbita moschata* (1) | Nov. 2009 | MED |  |
|  |  |  | *Solanum melongena* (2) | Nov. 2009 | MED |  |
|  |  |  | *Capsicum annuum* (2) | Nov. 2009 | MED |  |
| Ningbo Suburbs | 29°92'N | 121°57'E | *Capsicum annuum* (2) | Apr. 2009 | MED |  |
|  |  |  | *Cucumis sativus* (1) | Nov. 2009 | MEAM1, MED |  |
|  |  |  | *Brassica napus* (3) | Nov. 2009 | MEAM1, MED |  |
|  |  |  | *Phaseolus vulgaris* (4) | Nov. 2009 | MEAM1, MED |  |
|  |  |  | *Brassica campestris* ssp. *pekinensis* (3) | Nov. 2009 | MEAM1, MED |  |
|  |  |  | *Humulus japonicus* (9) | Nov. 2009 | MEAM1, MED |  |
| Shangyu, Shaoxing | 30°01'N | 120°90'E | *Brassica campestris* ssp. *pekinensis* (3) | Oct. 2009 | MEAM1, MED |  |
|  |  |  | *Ipomoea batatas* (5) | Oct. 2009 | MEAM1, MED |  |
|  |  |  | *Solanum melongena* (2) | Oct. 2009 | MEAM1, MED |  |
| Liuxia, Hangzhou | 30°24'N | 120°05'E | *Humulus japonicus* (9) | Jun. 2009 | Asia II 3 |  |
| Xiaoshan, Hangzhou | 30°29'N | 120°29'E | *Capsicum annuum* (2) | Mar. 2010 | MEAM1, MED |  |
| Qiaosi, Hangzhou | 30°35'N | 120°29'E | *Solanum lycopersicum* (2) | Feb. 2010 | MEAM1, MED |  |
|  |  |  | *Capsicum annuum* (2) | Feb. 2010 | MEAM1, MED |  |
|  |  |  | *Solanum melongena* (2) | Feb. 2010 | MEAM1, MED |  |
|  |  |  | *Ipomoea batatas* (5) | Nov. 2009 | MEAM1, MED |  |
|  |  |  | *Glycine max* (4) | Jul. 2009 | MEAM1 |  |
| Jiashan, Jiaxing | 30°84'N | 120°80'E | *Cucurbita pepo* (1) | Oct. 2009 | MEAM1, MED |  |
|  |  |  | [*Brassica oleracea*](http://en.wikipedia.org/wiki/Brassica_oleracea)var. *capitata* (3) | Oct. 2009 | MEAM1 |  |
|  |  |  | *Solanum melongena* (2) | Oct. 2009 | MEAM1, MED |  |
| Anji, Huzhou | 30°63'N | 119°73'E | *Capsicum annuum* (2) | Jun. 2009 | MED |  |
|  |  |  | *Solanum melongena* (2) | Jun. 2009 | MED |  |
|  |  |  | *Cucumis sativus* (1) | Jun. 2009 | MED |  |
| **(10) Sichuan province** | | |  |  |  |  |
| Mouzi town, Leshan | 29°36'N | 103°45'E | *Glycine max* (4) | Jul. 2009 | China 1 | HM137316 |
| Chunxi Road, Chengdu | 30°39'N | 104°04'E | *Euphorbia pulcherrima* (7) | Jul. 2009 | MEAM1  MED | HM137324, HM137331 |
| Maojiaqiao, Chengdu | 30°44'N | 103°54'E | *Glycine max* (4) | Jul. 2009 | China 1 | HM137315 |
| Tianfu Plaza, Chengdu | 30°66''N | 104°06'E | *Salvia coccinea* (8) | Jul. 2009 | MEAM1, MED |  |
| Chenkang, Mianyang | 31°39'N | 104°51'E | *Ipomoea batatas* (5) | Jul. 2009 | China 1 | HM137318 |
| **(11) Chongqing** | | |  |  |  |  |
| Guangmingbai, Beibei | 29°46'N | 106°22'E | *Glycine max* (4) | Jul. 2009 | China 1 | HM137341, HM137359 |
| Xinan University, Beibei | 29°81'N | 106°41'E | *Ipomoea batatas* (5) | Jul. 2009 | China 1 | HM137350, HM137351 |
| **(12) Hubei province** | | |  |  |  |  |
| Xiaochi, Huangmei | 29°76'N | 115°99'E | *Brassica campestris* ssp. *chinensis* (3) | Oct. 2009 | MED |  |
|  |  |  | *Ipomoea batatas* (5) | Oct. 2009 | MED |  |
|  |  |  | *Raphanus sativus* var. *longipinnatus* (3) | Oct. 2009 | MED |  |
|  |  |  | *Solanum melongena* (2) | Oct. 2009 | MED |  |
| Honghu Port, Honghu | 29°84'N | 113°42'E | *Solanum melongena* (2) | Aug. 2009 | MED |  |
| Niubutou, Honghu | 29°90'N | 113°55'E | *Gossypium hirsutum* (6) | Aug. 2009 | China 1  MED, Asia II 3 | HM137344 |
| Wuhu Farm, Wuhan | 30°73'N | 114°39'E | *Euphorbia pulcherrima* (7) | Aug. 2009 | MED |  |
| **(13) Anhui province** | | |  |  |  |  |
| MEAM1iyang, Huangshan | 29°91'N | 117°95'E | *Ipomoea batatas* (5) | Oct. 2009 | MED | HM137320 |
| Madao, Huangshan | 29°93'N | 117°94'E | *Cucumis sativus* (1) | Oct. 2009 | MED |  |
| Hefei suburbs | 31°92'N | 117°14'E | *Salvia coccinea* (8) | Oct. 2009 | MEAM1 |  |
| Sanshigang, Hefei | 31°92'N | 117°15'E | *Raphanus sativus* var. *longipinnatus* (3) | Oct. 2009 | MED |  |
| Paifang, Hefei | 31°95'N | 117°48'E | *Capsicum annuum* (2) | Oct. 2009 | MED |  |
|  |  |  | *Cucumis sativus* (1) | Oct. 2009 | MED |  |
|  |  |  | *Solanum melongena* (2) | Oct. 2009 | MED |  |
|  |  |  | *Solanum lycopersicum* (2) | Oct. 2009 | MED |  |
| **(14) Shanghai** | | |  |  |  |  |
| Chongming Island | 31°50'N | 121°80'E | *Brassica oleracea* var. *capitata* (3) | Nov. 2009 | MEAM1 |  |
|  |  |  | *Capsicum annuum* (2) | Nov. 2009 | MED |  |
| **(15) Jiangsu province** | | |  |  |  |  |
| Nanjing suburbs | 32°02'N | 118°54'E | *Solanum lycopersicum* (2) | Sep. 2009 | MED |  |
|  |  |  | *Solanum melongena* (2) | Sep. 2009 | MED |  |
|  |  |  | *Brassica oleracea* var. *capitata* (3) | Sep. 2009 | MED |  |
|  |  |  | *Gossypium hirsutum* (6) | Sep. 2009 | MED |  |
| Shagang, Huai’an | 32°59'N | 118°29'E | *Solanum melongena* (2) | Sep. 2009 | MED |  |
|  |  |  | *Gossypium hirsutum* (6) | Sep. 2009 | MED |  |
|  |  |  | *Cucumis sativus* (1) | Sep. 2009 | MED |  |
| **(16) Henan province** | | |  |  |  |  |
| Luoyang Suburbs | 34°46'N | 112°46'E | *Ipomoea batatas* (5) | Sep. 2009 | MED | HM137321 |
| Li Village, Luoyang | 34°59'N | 112°58'E | *Solanum melongena* (2) | Sep. 2009 | MEAM1 |  |
|  |  |  | *Cucumis sativus* (1) | Sep. 2009 | MEAM1 |  |
| Lou Village, Luoyang | 34°66'N | 112°51'E | *Brassica oleracea* var. *capitat* (3) | Sep. 2009 | MEAM1 |  |
|  |  |  | *Brassica oleracea* var. *botrytis* (3) | Sep. 2009 | MEAM1 |  |
|  |  |  | *Gossypium hirsutum* (6) | Sep. 2009 | MEAM1 |  |
| Zhenzhou Suburbs | 34°78'N | 113°66'E | *Nicotiana tabacum* (2) | Sep. 2009 | MEAM1, MED |  |
|  |  |  | *Glycine max* (4) | Sep. 2009 | MEAM1, MED |  |
|  |  |  | *Cucumis sativus* (1) | Sep. 2009 | MEAM1 |  |
|  |  |  | *Solanum melongena* (2) | Sep. 2009 | MED |  |
|  |  |  | *Solanum lycopersicum* (2) | Sep. 2009 | MEAM1, MED |  |
|  |  |  | *Capsicum annuum* (2) | Sep. 2009 | MEAM1, MED |  |
| Chenzhai Flowers Market, Zhenzhou | 34°82'N | 113°65'E | *Euphorbia pulcherrima* (7) | Sep. 2009 | MEAM1, MED |  |
| Huiji, Zhenzhou | 34°86'N | 113°61'E | *Brassica oleracea* var. *capitata* (3) | Sep. 2009 | MEAM1, MED |  |
|  |  |  | *Solanum lycopersicum* (2) | Sep. 2009 | MEAM1, MED |  |
|  |  |  | *Phaseolus vulgaris* (4) | Sep. 2009 | MEAM1 |  |
| Zhuoshui, Xinxiang | 35°47'N | 113°75'E | *Phaseolus vulgaris* (4) | Sep. 2009 | MEAM1, MED |  |
|  |  |  | *Brassica campestris* ssp. *pekinensis* (3) | Sep. 2009 | MEAM1, MED |  |
|  |  |  | *Ipomoea batatas* (5) | Sep. 2009 | MEAM1, MED |  |

* Locations are listed from south to north, first the provinces and then the localities within each province.

† In all 29 species of host plants in 11 families, figures in parentheses indicate the names of the families: (1) Cucurbitaceae, (2) Solanaceae, (3) Brassicaceae, (4) Leguminosae, (5) Convolvulaceae, (6) Malvaceae, (7) Euphorbiaceae, (8) Lamiaceae, (9) Cannabaceae, (10) Piperaceae, (11) Verbenaceae.
